# Low-dose Radiation Therapy (LDRT) in Managing Osteoarthritis: A Comprehensive Review

**DOI:** 10.1016/j.curtheres.2025.100777

**Published:** 2025-02-12

**Authors:** Armin Hoveidaei, Mehdi Karimi, Amirhossein Salmannezhad, Yasaman Tavakoli, Seyed Pouya Taghavi, Amir Human Hoveidaei

**Affiliations:** 1 Students’ Scientific Research Center, Exceptional Talents Development Center, Tehran University of Medical Sciences, Tehran, Iran; 2 Faculty of Medicine, Bogomolets National Medical University (NMU), Kyiv, Ukraine; 3 Student Research Committee, Qazvin University of Medical Sciences, Qazvin, Iran; 4 Student Research Committee, Department of Medicine, Mazandaran University of Medical Science, Sari, Iran; 5 Student Research Committee, Kashan University of Medical Sciences, Kashan, Iran; 6 International Center for Limb Lengthening, Rubin Institute for Advanced Orthopedics, Sinai Hospital of Baltimore, Baltimore, Maryland

**Keywords:** Osteoarthritis, Radiation therapy, Radiotherapy, Radiation, Review

## Abstract

Osteoarthritis (OA) is the most common degenerative arthropathy, impacting the quality of life for millions worldwide. It typically presents with chronic pain, stiffness, and reduced mobility in the affected joints. Nonsurgical treatments like physiotherapy or pharmacotherapy may provide limited relief and may have adverse effects and complications. Recently, low-dose radiation therapy (LDRT) has emerged as a potential alternative for managing OA, utilizing its anti-inflammatory effects. LDRT's anti-inflammatory effects involve modulating immune responses, reducing pro-inflammatory cytokines, and inducing apoptosis in inflammatory cells. Clinical studies show varying degrees of symptom relief, with some patients experiencing pain reduction and improved joint mobility while others show minimal response. The variability in LDRT treatment designs, radiation dosages, and patient populations complicates standardized treatment protocols and raises concerns about potential carcinogenic risks. Despite these issues, LDRT shows promise as an alternative to other OA treatments, especially for patients who don't respond to other treatments. This review aims to provide updated information on the effectiveness, mechanisms, and safety of LDRT in treating OA. We reviewed the literature of studies on the safety and efficacy of LDRT on affected joints by OA, its biological effects, potential therapeutic and adverse effects, application and contraindications, clinical outcomes, and clinical evidence in subjects with OA.

## Graphical Abstract

**Figure:**
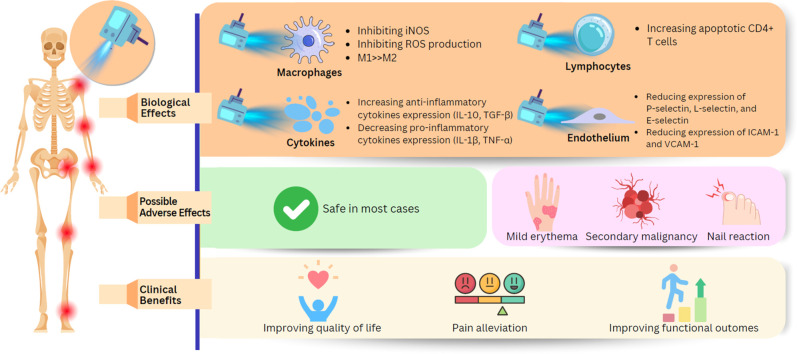

## Introduction

Osteoarthritis (OA) is the most common form of arthritis,^1^ affecting millions of people around the world and is a leading cause of disability, particularly among the elderly.^2^ The most common treatments for OA are pharmacological approaches, physical therapy, and, in severe cases, surgery. However, these treatments fail to provide long-term relief, particularly for patients with severe or refractory symptoms.^3^^,^^4^

Low-dose radiation therapy (LDRT), traditionally employed in oncology, uses radiation at significantly lower levels than those used to treat cancer. In recent years, LDRT has received interest as a potential treatment for OA.^5^^,^^6^ Its anti-inflammatory properties and ability to control cellular processes make it an intriguing candidate for treating OA-related inflammation and pain.^7^ However, many parts of the world underutilize and underexplore LDRT, despite its adoption in several European countries.

Given the increasing interest in alternative therapies for OA and the growing body of research on LDRT, this review seeks to provide a literature review of the existing evidence on LDRT in OA. It investigates the biological mechanisms that underpin LDRT's therapeutic effects, assesses its clinical efficacy, and analyzes the possible benefits and limitations of incorporating this treatment into OA management methods. This review examines the existing evidence to define LDRT's role in OA care and recommends areas for future investigation.

## Method

A comprehensive literature search was conducted using MeSH terms and query terms including “Low-dose Radiation Therapy” OR “LDRT” OR “Radiation Therapy” OR “Radiotherapy” AND “Osteoarthritis” OR “degenerative joint diseases” OR “Arthrosis”, across PubMed and ISI Web of Science databases. Additionally, a manual search was performed using Google Scholar to identify relevant studies published within the last 10 years, focusing on the most recent and updated articles related to the use of LDRT for managing osteoarthritis. The search was restricted to studies that provided evidence of the safety, efficacy, and mechanisms of LDRT in OA treatment. Articles that did not meet the inclusion criteria, such as those unrelated to OA or not focusing on LDRT, were excluded. Relevant studies were selected based on their methodological quality and clinical relevance to provide a comprehensive overview of LDRT's role in managing osteoarthritis.

## An Overview of Osteoarthritis

OA is a chronic, degenerative joint illness defined by the destruction of articular cartilage, which causes pain, stiffness, and decreased mobility.^1^^,^^8^ Among the musculoskeletal disorders (MSDs), OA is a major contributor to the number of years spent disabled. Because OA is more common among the elderly (about 70% are over the age of 55), its global incidence is predicted to rise as populations age. The average beginning is in the late 40s to mid-50s, but OA can also affect younger people, such as athletes and those who have had joint injury or trauma. Women account for approximately 60% of those living with OA.^9^^,^^10^

It is proven that mechanical, genetic, and metabolic factors influence the development of OA. Mechanical stress, such as femoroacetabular impingement, is a common cause of secondary OA in young adults, leading to damage to the capsular-labrum complex and cartilage over time.^11^^,^^12^ In addition, it has been shown that genetic predisposition, including mutations like NOV/CCN3 and a family history of OA, can contribute to the pathogenesis of the disease.^13^ Metabolic dysfunction, like SIRT5 deficiency hurting chondrocytes and sustained Akt signaling causing oxidative stress, also plays a part in the progression of OA, especially as people age.^14^

The risk of developing osteoarthritis increases with age, and factors such as female sex, joint injuries, and repeated stress on the joints can contribute to its onset.^15^ Obesity is a significant lifestyle-related factor, particularly higher body mass index (BMI). Certain medical conditions, including diabetes, hypertension, joint injuries, and previous joint surgeries, also elevate the risk.^16^^,^^17^ Additionally, joint-specific abnormalities such as residual hip dysplasia (RHD), Perthes disease, slipped capital femoral epiphysis (SCFE), isolated malrotation of the hip joint, and reduced femoral head-neck offset further increase the likelihood of developing OA.^11^^,^^18^

The diagnosis of OA requires a comprehensive strategy that includes patient history, physical examination, imaging, and modern diagnostic technologies. A detailed history (Hx) evaluates important aspects such as age, symptoms, including persistent pain and stiffness, and risk factors, including obesity and past joint injuries. The physical examination (PE) evaluates the joints for edema, pain, crepitus, misalignment, and muscular weakness.^19^ X-rays are the usual imaging method for diagnosing OA, revealing characteristics such as joint space narrowing, osteophytes, and subchondral sclerosis, which are frequently graded on the Kellgren-Lawrence scale. However, X-ray results may not necessarily be associated with symptoms, particularly in early OA.^20^ Advanced imaging, such as magnetic resonance imaging (MRI) and computed tomography (CT), can detect early cartilage changes and complicated bone structures, whereas arthroscopy allows for direct observation of joint tissues.^21^^,^^22^

Conventional therapies for OA commonly recommend intra-articular injections of hyaluronic acid (IA-HA) and corticosteroids (IA-CS), while platelet-rich plasma (PRP) injections are gaining attention with fewer guidelines supporting them.^23^^,^^24^ There is a growing emphasis on early OA detection and intervention, aiming to implement disease-modifying strategies that could delay or prevent arthroplasty. Emerging treatments, such as disease-modifying OA drugs, chondrocyte implantation, and stem cell therapy, target OA in its early stages.^25^

## Low-Dose Radiation Therapy (LDRT)

LDRT has been investigated as a treatment for various MSDs, including OA. For decades, it has primarily been used to treat benign inflammatory and degenerative disorders, such as epicondylitis and plantar fasciitis. By employing low doses of radiation, LDRT aims to achieve anti-inflammatory effects and pain relief, positioning it as a potential noninvasive option for managing conditions like OA.^26^^,^^27^

It is unclear how LDRT alleviates OA symptoms. However, it is thought to include the regulation of inflammatory processes. LDRT may inhibit the generation of pro-inflammatory cytokines and other inflammatory mediators, reducing pain and inflammation in afflicted joints.^28^

LDRT presents an alternative for patients who may not respond adequately to conventional treatments, such as NSAIDs or corticosteroid injections, offering pain relief without the systemic side effects associated with these medications. While conventional therapies primarily focus on symptom management, LDRT targets the underlying inflammatory processes, potentially providing more sustained relief.^5^^,^^29^ Additionally, LDRT is generally well-tolerated and carries a lower risk of adverse effects compared to higher-dose radiation therapies used in oncology; however, further long-term safety data are still needed to fully assess its risk profile.^30^

## Biological Effects of LDRT

According to current literature, the exact radiobiological mechanisms of LDRT are not yet well understood. The mechanism of action of LDRT is primarily based on immunomodulatory processes, which results in anti-inflammatory effects. LDRT can potentially regulate anti- and proinflammatory cytokine production and inflammation mediator cells’ function.^31^^,^^32^

### Mechanism of action

Synovial cells and resident macrophages are responsible for cartilage matrix degradation and inflammatory environment by producing proinflammatory cytokines, reactive oxygen species (ROS),^33^^,^^34^ and metalloproteinases (MMPs).^35^, ^36^, ^37^ An experimental study has shown that 2 Gy of LDRT significantly induces apoptosis of fibroblast-like synoviocytes.^38^ In addition to the aforementioned pathways macrophages in the pathogenesis of OA, inducible nitric oxide synthesis (iNOS) has been suggested to be an inflammatory mediator, facilitating cartilage degradation and cellular damage.^39^ It is reported that LDRT inhibited iNOS in stimulated macrophages.^40^ Kim et al, demonstrated a reduction of MMP-13 and, conversely, an elevation of collagen type 2 (COL2) following LDRT in an OA model.^41^ Furthermore, radiotherapy with doses lower than 1 Gy causes the transformation of pro-inflammatory M1 phenotype macrophages into M2 phenotype, which acts as an anti-inflammatory component.^42^

Leukocytes are also modified by LDRT in various ways. It has been indicated that recruitment of leukocytes was optimally inhibited at 0.3 Gy.^43^ In a clinical study, the serum levels of leukocytes were constant following six fractions of LDRT with a dose of 0.5 Gy in each fraction.^44^ Nevertheless, the number of some cell types was changed as follows: increased levels of basophile, eosinophile, and plasmacytoid dendritic cells and decreased levels of B lymphocytes. LDRT exposure could enhance the apoptosis of the leukocytes.^44^^,^^45^ Another study demonstrated elevated apoptotic CD4+ T cells.^38^ Moreover, LDRT has been reported to restrict the migration of leukocytes through the downregulation of adhesion molecule expression (eg, selectin, ICAM, and VCAM).^46^^,^^47^ In an experimental investigation, IL-17A and CD8+ T-cells serum levels were significantly diminished in OA-induced murine models.^7^ Production of pro-inflammatory and inflammatory cytokines can potentially be inhibited after irradiation.^43^^,^^48^, ^49^, ^50^ However, TGF-β1, which acts as an anti-inflammatory cytokine here, has been observed to be produced in higher quantities in the blood.^51^ This was supported by a previous study, explaining that LDRT includes endothelial cells as a source of TGF-β1.^52^

Additionally, Growth Differentiation Factor-15 (GDF-15) has been recently found to be a measure of chronic pain in MSDs and the risk of cardiovascular disease in patients with CMP.^53^ Also, It has been particularly investigated on OA individuals, highlighting that GDF-15 can serve as a predictor of long-term mortality independently of other comorbidities.^54^ Kim et al, observed no long-term increase in GDF-15 expression or serum level; however, a transient increase in GDF-15 was noted, which resulted in mitochondrial regulation, mediating anti-inflammatory effects, and subsequently attenuated the progression of OA.^41^

### Other biological effects

Radiation treatment with lower doses has been found to result in continuous reduction in bone mass by inducing osteoclastogenesis, leading to reduced bone density.^55^, ^56^, ^57^ In a study by Deloch et al, following exposure to a 0.5 Gy dose, a subtle transition from CD8+ to CD4+ T cells, a slight reduction in osteoclastic bone resorption, and a modest increase in bone marrow cell death rates were observed in healthy joints. They claimed that LDRT caused no detrimental effect on healthy joints.^58^

Although several studies have shown that articular cartilage is insensitive to radiation,^59^, ^60^, ^61^ there is a lack of coherence due to the diverse methods used in the studies, including variations in dosage and dose rate. In this context, we discuss the biological effects of LDRT on articular cartilage, focusing on studies that employed methods similar to OA LDRT. In an experimental study on 40 knees of 20 rabbits, no substantial differences were found in biomechanical properties up to 12 weeks after receiving 1.0 Gy fractions and a total dose of 5 Gy.^62^ Toda et al, reported dissipation of the mitochondrial membrane potential as a representation of apoptosis in degenerated cartilage after x-ray irradiation but a remarkable resistance of nondegenerated cartilage against apoptosis.^63^ Overall, current evidence tends to show that LDRT has beneficial or neutral effects on articular cartilage rather than detrimental ones.

## Therapeutic and Adverse Effects of LDRT on OA

LDRT is mostly considered as a safe and well-tolerated treatment, with only a few reports of mild adverse effects.^64^ Only nonserious adverse events (NSAE), such as mild erythema, were reported.^65^ In a randomized controlled trial, no adverse effects were observed in patients who received a total dose of 3.0 Gy and two single 0.5 Gy fractions administered weekly at the 12-month follow-up.^66^ Another long-term evaluation of LDRT showed that single doses of 0.5 Gy are a safe option.^67^ Patients with hand or knee OA experienced skin reactions, nail reactions, and fatigue after LDRT, with similar numbers to sham group.^68^ However, patients with hand OA were observed to present a comparable higher number of nail reactions than the sham group. Also, no serious adverse effects were found to be related to the irradiation trial.^68^

As patients have been exposed to ionizing radiation during several sessions of LDRT, the risk of secondary malignancy should be discussed. Studies have shown that the minimum dose needed for a high risk of secondary malignancy is higher than 2.5 Gy at the primary radiation field, which is the usual dose of LDRT in OA (3–6 Gy for total dose).^69^ A large study involving 27,011 Chinese medical X-ray workers indicated that red bone marrow dose has a positive relationship with non-CLL (chronic lymphocytic leukemia) leukemia incidence, highlighting a higher risk of leukemia in subjects exposed to low dose ionizing radiation compared to those who were not.^70^ A meta-analysis revealed that breast cancer risk is significantly elevated in predisposed women who received low-dose radiation before 20 or have more than five exposures.^71^ However, it should be noted that the patients had direct exposure to radiation, such as through chest X-ray or mammography. In this regard, there are two important factors to be considered: 1) the age range of the targeted population for treating OA with LDRT is beyond that to be concerned as malignancies require decades to progress, and 2) targeted regions for irradiation are not mostly nearby organs at high risk of malignancies. The tissues residing in joints are not susceptible to cancer.^72^ LDRT is performed locally and has no contact with the whole body, suggested to be at low risk of secondary malignancy.

## Application and Contraindications of LDRT

LDRT is not currently recommended as first-line treatment, but it is a viable choice for OA patients who did not respond to earlier medical therapies. Additionally, some cases requiring surgical intervention may preoperatively benefit from the radiotherapy, depending on the physician's discretion. According to the latest update of the German Society of Radiation Therapy and Oncology (DEGRO) guideline, LDRT is suggested as a higher level of recommendation for treating knee OA (category B) than hip, hand, and shoulder (category C).^73^ Category B is defined as “Should be carried out” and was proposed based on evidence level II or III. On the other hand, category C is described as “Can be carried out” and is supported by evidence level IV. DEGRO guidelines have not recommended any indication of LDRT for ankle OA. However, it has been recently indicated that LDRT can potentially alleviate the pain and improve joint mobility.^74^ More studies are needed to establish the effectiveness of LDRT on ankle OA.

There might be some concerns about RT exposure in OA patients. For instance, due to the low exposure, there is low concern about fetus radiation during pregnancy. Applying ionizing radiation for shoulder may subject the patients to a risk for cardiovascular problems, due to its nearby irradiation. Patients with breast cancer had an excess risk of cardiovascular diseases, exposure to 20 fractions of 2 Gy,^75^ confirmed the risk of cardiovascular diseases following LDRT by other studies.^76^ However, there is no evidence denoting any cardiac risk after LDRT for OA patients. In addition to the shoulder, radiotherapy targeting the hip joint may raise concerns about the potential risk of cancer in nearby sensitive organs, such as the prostate, uterus, or ovaries. However, to date, no definite contraindication was documented, including pregnancy, underlying conditions (eg, diabetes), active infection (eg, septic arthritis), cardiovascular diseases, and previous radiation therapy (especially high doses) or ongoing radiotherapy.

## Discussion and Clinical Evidence

So far, 25 studies evaluated the effects of LDRT in OA patients. According to Table 1, twelve retrospective studies with a level of evidence of III validated the pain relief and positive influence on joint function by LDRT. Furthermore, six prospective studies with level II evidence found that LDRT reduced the numerical rating scale (NRS), visual analog scale (VAS), and Von Pannwitz Score (VPS) in OA patients. However, five randomized clinical trial studies with a level of evidence of I questioned the efficacy of LDRT on OA. Two observational studies with a level of evidence III revealed a decrease in VAS score following LDRT. The lowest radiation dose was 0.05 Gy per fraction for a total dose of 0.3 Gy, while the highest dose was 1 Gy per fraction for a total of 6 Gy. Follow-ups range from 8 to 52 weeks. The total number of samples ranged from 16 to 4544. All research was carried out in Europe, including 19 in Germany.

**Table 1:** Main clinical evidence and review.

Study/year	Country	Study design / Sample size	Site of OA	Total dose /dose fraction (Gy)	Pain scoring	Follow-up	Treatment device	Outcomes
Niewald, M., et al.^81^	Germany	RCT (n = 244)	Hand, finger, knee	3.0 /0.5 or 0.3 /0.05	VAS	12 months	NR	Markedly improved: 41% Improved: 20% No significant differences between treatment arms.
Weissmann, T., et al.^82^	Germany	Retrospective (n = 196)	Foot, ankle	3.0 /0.5 or 6.0 /1.0	Subjective patient-reported pain reduction	3 and 6 months	Orthovoltage	The response rate by 6 months was 75%. 37% of cases experienced pain reductions of 80%–100%.
Niewald, M., et al.^83^	Germany	RCT (n = 229)	Hand, knee	3.0 /0.5 or 0.3 /0.05	VAS	3 to 12 months	Linac	There was no meaningful difference between treatment arms.
Álvarez, B., et al.^84^	Spain	Prospective (n = 100)	Hand	6.0 /1.0 or 3.0 /0.5	VAS	10.5 months (median)	Linac	After three, six, and twelve months, 94% reported pain relief, with a significant decrease in VAS.
Rühle, A., et al.^85^	Germany	Retrospective (n = 1185)	Multijoint	6.0 /1.0 or 3.0 /0.5	NRS	8 weeks after reirradiation	Linac	NRS significantly decreased.
Donaubauer, A.-J., et al.^44^	Germany	Prospective (n = 125)	Multijoint	3.0 /0.5	VAS	6 months	Orthovoltage	The mean VAS was reduced from 6.5 to 3.8.
van den Ende, C. H. M., et al.^68^	The Netherlands	RCT (n = 56)	Knee, hand	6.0 /1.0 Or sham irradiation	OMERACT- OARSI criteria	6 and 12 months	Linac	There was no meaningful difference between treatment arms. (respond rate = sham:44% vs LDRT:52%)
Rogers, S., et al.^86^	Switzerland	Prospective (n = 99)	Fingers	4.0 /0.5	VAS	12 months	Orthovoltage	VAS decreased by 3.0 (median) during physical activity
Hautmann, M. G., et al.^87^	Germany	Retrospective (n = 295)	Multijoint	3.0 /0.5, 5.0 /1, 1.0 /1.0, 5.0 /0.5, 1.5 /0.5, or 6.0 /1.0	NRS	19 months (median)	Linac	Mean NRS was reduced from 5 to 3.
Hautmann, M. G., et al.^88^	Germany	Retrospective (n = 66)	Ankle and tarsal joints	3.0 /0.5, 5.0 /1, or 6.0 /1.0	NRS	31 months (median)	Linac	The median NRS was 7 before radiation, 5 after 6 and 12 weeks, and 4 after 12 months. Improvement of joint mobility was reported in 56.7% of the cases.
Donaubauer, A. J., et al.^89^	Germany	Retrospective (n = 483)	Fingers and thumb	3.0 /0.5 or 6.0 /1.0	Subjective patient- reported pain reduction	12 and 24 weeks	Orthovoltage	At the end of RT, 70% of patients reported reduced pain.
Mahler, E. A. M., et al.^90^	The Netherlands	RCT (n = 55)	knee	sham irradiation or 6.0 /1.0	OMERACT-OARSI criteria	3 months	Linac	There was no meaningful difference between treatment arms. (respond rate = sham:43% vs LDRT:44%)
Koc, B. B., et al.^91^	The Netherlands	Prospective (n = 16)	Knee and hip	6.0 /1.0	NRS	52 weeks	Linac	The response rate to pain reduction was 50% at 6 weeks and 25% at 52 weeks.
Juniku, N., et al.^92^	Germany	Retrospective (n = 598)	Multijoint	3.0 /0.5, Or 5.0 /0.5	VAS	38 months (median)	Linac	62.4% of patients responded as (VAS 0–2) at 38 months; The mean VAS was reduced from 7.0 to 1.0.
Hautmann, M. G., et al.^93^	Germany	Retrospective (n = 217)	Reirradiated Multijoint	3.0 /0.5, or 2.0 /0.5	NRS	25 months (median)	Linac	Free pain or a little pain after 12 months: 25% The mean NRS was reduced from 6.0 to 3.0.
Minten, M. J. M., et al.^94^	The Netherlands	RCT (n = 56)	Hand	sham irradiation or 6.0 /1.0	OMERACT-OARSI criteria	3 months	Linac	There was no meaningful difference between treatment arms. (respond rate = sham: 36% vs LDRT: 29%)
Micke, O., et al.^95^	Germany	Prospective (n = 703)	Multijoint	6.0 /0.5, or 6.0 /1.0	VAS and VPS	33 months (median)	Linac orthovoltage	The mean VAS was reduced from 7.0 to 4.5. The response rate to pain reduction was 37.6% at the end of the RT and 58.4% at 33 mo.
Micke, O., et al.^96^	Germany	Prospective (n = 166)	Multijoint	6.0 /0.5, or 6.0 /1.0	VAS and VPS	29 months (median)	Linac orthovoltage	The mean VAS was reduced from 6.38 to 4.49. The response rate to pain reduction was 37.3% at the end of the LDRT and 49.6% at 29 mo.
Kaltenborn, A., et al.^97^	Germany	Retrospective (n = 101)	Thumb	6.0 /1.0	Subjective patient-reported pain reduction	3 and 12 months	Linac	The response rate to pain reduction was 63% at 3 months and 70.3% at 12 mo.
Ott, O. J., et al.^98^	Germany	Observational (n = 112)	Joint with Achillodynia	6.0/0.5–1	VAS	24 months	Orthovoltage	Immediate pain relief was observed in 84% of cases, while late pain relief was reported in 95%..
Ott, O., et al.^99^	Germany	Observational (n = 284)	Painful shoulder syndrome	6.0/0.5–1	VAS	35 months	Orthovoltage	Immediate pain relief was observed in 83% of cases, while late pain relief was reported in 82%.
Keller, S., et al.^100^	Germany	Retrospective (n = 1037)	Knee	0.5–10 /0.5–1.5	VPS	2 months	Linac orthovoltage Cs-137	79.3% of the patients responded to the LDRT.
Mücke, R., et al.^101^	Germany	Retrospective (n = 4544)	Joint with gonarthrosis	3.0–12.0/0.25–3.0	Subjective patient- reported pain reduction	3 and 12 months	Orthovoltage unit, cobalt-60, or Linac	Immediate pain relief was reported in 60% of cases. Late pain relief was reported in 40% of the cases.
Ruppert, R., et al.^102^	Germany	Retrospective (n = 73)	Multijoint	6.0/0.5	OMERACT- OARSI criteria	48 months	Orthovoltage	63% of the patients responded to the LDRT.
Glatzel, M., et al.^103^	Germany	Retrospective (n = 114)	Joint with gonarthrosis	3.0–6.0/0.5–1	NR	29 months	NR	Pain relief was reported in 68% of the cases.

LDRT = low-dose radiation therapy; linac = linear accelerator; OMERACT-OARSI = Outcome Measures in Rheumatology−Osteoarthritis Research Society International; NRS = numerical rating scale; NR: NON-REPORTED; RCT: Randomized clinical trial; RT = radiation therapy; VAS = visual analog scale; VPS = von pannwitz score.

The lowest recorded response to LDRT in OA patients was 25%, with the greatest reported response being around 100%. Eleven studies used multijoint samples, and all of them reported pain reduction after LDRT at various follow-ups. Overall, eight studies used LDRT on the hands or fingers, and the results varied depending on the type of study and radiation dose. Six studies performed LDRT on the knee, and the outcomes varied depending on the type of study and radiation intensity used. Two retrospective investigations focused specifically on LDRT for the foot and ankle, and both found pain reduction based on NRS and patient reports. Only one study used LDRT on the hip and observed pain reduction after 6 and 52 weeks. One observational trial used LDRT for painful shoulder syndrome, and 83% of patients reported pain reduction after 35 weeks of follow-up.

Table 1 summarizes the most recent literature on OA therapy with LDRT.

If OA-related pain and movement limits are not appropriately managed, various negative health consequences might arise. As a result, patients with OA may pay large medical expenses each year, placing a socioeconomic load on the national medical system. To promote LDRT in countries where it is not commonly used, convincing medical evidence should be supplied.^77^ In Germany in 2014, approximately 15,000 people underwent radiotherapy.^78^ According to the German Society of Radiation Oncology guidelines, LDRT can be used to treat painful OA of both large joints (knees, hips, and shoulders) and small joints (wrist, fingers, thumbs, ankle, and foot).^79^ As Table 1 shows, the use of LDRT in Europe has increased in recent years, but it has yet to reach the United States. Dove et al, (2022) claim that LDRT is a low-cost, noninvasive treatment with few side effects, citing trials showing moderate to long-term pain reduction and better mobility.^5^ According to Javadinia et al, (2021), multiple single-arm trials have shown that LDRT has benefits in the management of OA, including pain reduction and functional improvement. They also propose a mechanism that involves the stimulation of antioxidant responses.^80^ Given the inconsistent data, LDRT could be a potential treatment for OA.

## Conclusion

In conclusion, LDRT emerges as a promising alternative treatment for managing OA, offering significant pain relief and functional improvement with minimal adverse effects. While the exact biological mechanisms remain under investigation, LDRT's anti-inflammatory properties and its potential to modulate immune responses suggest it could serve as a valuable treatment option, particularly for patients who do not respond to conventional therapies. Despite the positive clinical outcomes observed in several studies, variability in treatment protocols and patient responses calls for further research. Large-scale, randomized clinical trials with standardized dosages are needed to solidify LDRT's efficacy and safety profile for widespread use in OA management.

## Declaration of competing interest

The authors declare that they have no known competing financial interests or personal relationships that could have appeared to influence the work reported in this paper.
